# Impact of Atomic Layer-Deposited Hydroxyapatite-Coated Titanium on Expression of Focal Adhesion Molecules of Human Gingival Fibroblasts

**DOI:** 10.3390/nano15120887

**Published:** 2025-06-08

**Authors:** Nagat Areid, Faleh Abushahba, Sini Riivari, Elisa Närvä, Elina Kylmäoja, Mikko Ritala, Juha Tuukkanen, Pekka K. Vallittu, Timo O. Närhi

**Affiliations:** 1 Department of Prosthetic Dentistry and Stomatognathic Physiology, Institute of Dentistry, University of Turku, FI-20520 Turku, Finland; nmaare@utu.fi (N.A.); sini.k.riivari@utu.fi (S.R.); timnar@utu.fi (T.O.N.); 2 Department of Restorative Dentistry and Periodontology, Faculty of Dentistry, Libyan International Medical University (LIMU), Benghazi 339P+62Q, Libya; 3 Department of Biomaterials Science and Turku Clinical Biomaterials Center—TCBC, Institute of Dentistry, University of Turku, FI-20520 Turku, Finland; pekval@utu.fi; 4 Institute of Biomedicine and FICAN West Cancer Centre Laboratory, University of Turku and Turku University Hospital, FI-20520 Turku, Finland; elisa.narva@utu.fi; 5 Department of Anatomy and Cell Biology, Research Unit of Translational Medicine, Medical Research Center, University of Oulu, FI-90014 Oulu, Finland; elina.kylmaoja@oulu.fi (E.K.); kjuhak.tuukkanen@gmail.com (J.T.); 6 Department of Chemistry, University of Helsinki, FI-00014 Helsinki, Finland; mikko.ritala@helsinki.fi; 7 The Wellbeing Service County Southwest Finland, FI-20521 Turku, Finland

**Keywords:** ALD, cell adhesion, dental implant, gingival tissue, implant surface, titanium, focal adhesion

## Abstract

This study investigated the impact of the nanocrystalline atomic layer-deposited hydroxyapatite (ALD-HA) coating of titanium (Ti) surface on the attachment and proliferation of human gingival fibroblasts (HGFs). Ti discs were divided into ALD-HA-coated and non-coated (NC) controls. HGFs were harvested from gingival biopsies of patients subjected to extraction of their third molar. The cells were cultivated on the Ti discs for 2 and 24 h to evaluate the initial cell attachment using confocal microscopy. Spreading of cells and the signals of focal adhesion proteins were measured. Moreover, the adhesion proteins vinculin and paxillin expression levels were evaluated using Western blot after 3 d of cultivation. In addition, the proliferation of HGF was assessed by cultivating the cells on Ti discs for 1, 3, and 7 d. Fibroblast spreading was significantly greater on ALD-HA surfaces than on NC surfaces after 2 h (*p* < 0.001). In addition, the signals of vinculin and paxillin were significantly higher on the ALD-HA than on the NC surfaces at 2 and 24 h. The confocal microscope analysis also revealed significantly higher expression of focal adhesion molecules on ALD-HA surfaces at both time points. Furthermore, the cell proliferation rate was significantly higher at d 3 (*p* = 0.022) and d 7 (*p* < 0.001) on the ALD-HA compared to the NC surfaces. These findings indicate that ALD-HA coating enhances focal adhesion formation, cell spreading, and proliferation on Ti surfaces, suggesting its potential to improve gingival tissue attachment to Ti implant surfaces.

## 1. Introduction

The risk of infection is significantly high in all medical devices that penetrate the skin or mucosa, including oral implants [1]. In dental implants, the peri-implant mucosa is composed of epithelium as well as connective tissue attachment. These components share some clinical and histological similarities with natural teeth [2]. However, key differences lie in cellular composition and fiber orientation [3,4]. In dental implants, the peri-implant connective tissue fibers consist of a dense collagen fiber network. These fibers originate from the bone and run parallel to the implant or abutment surface without showing any evidence of insertion into the implant surface [3,5], unlike in natural teeth, where collagen fibers insert perpendicularly into the root cementum. The structural arrangement of connective tissue around dental implants makes them susceptible to epithelial downgrowth, allows bacterial infiltration into peri-implant tissues, and increases the risk of infection [6,7]. Therefore, establishing proper adhesion between the implant and surrounding tissue at the exit site can create an effective seal to control microbial infiltration into the peri-implant area.

Different surface modifications have been employed to achieve a bioactive coating on implant surfaces, thereby enhancing both the biological and biomechanical properties. One of these coatings is the hydroxyapatite coating, which has been shown to enhance initial healing and promote bone formation [8]. Various techniques have been developed to apply hydroxyapatite (HA) coatings to titanium (Ti) implants, such as sol-gel [9], pulsed laser deposition [10], magnetron sputtering [11], electrophoretic deposition [12], and plasma spraying [10]. Among these, plasma spraying of Ti implants has been extensively studied, with both in vivo evidence [13] and clinical data [14] supporting its strong osteoconductive properties. This method is cost-effective for producing coatings with thicknesses ranging from 30 to 200 µm [15]. However, plasma-sprayed coatings often exhibit low cohesive strength [16] and are applied at high temperatures, leading to inhomogeneities in chemical composition and crystal structure, as well as flaking, brittleness, and delamination [17,18]. Atomic layer deposition (ALD) is a method of functionalizing Ti surfaces to improve their chemical and physical properties by depositing a nanoscale coating with controlled thickness and good adhesion strength [19]. Earlier research indicated that ALD can produce a uniform HA layer of 300 nm thick with a plate-like crystal structure [20]. X-ray diffraction (XRD) analysis of the coating showed characteristic diffraction peaks corresponding to the HA standard reference pattern (JCPDS no. 09-0432) [20]. In addition, the ALD enables the coating of complex three-dimensional surfaces with a controlled film thickness and a good adhesion strength (6.71 Mpa) [20,21]. Therefore, this nano-coating could reduce the risks associated with the HA layer produced by conventional methods.

The surface chemistry influences not only osseointegration but also soft tissue integration [22,23,24]. Past research conducted by the authors has indicated that atomic layer-deposited HA (ALD-HA)-coated Ti surfaces support blood coagulation and platelet adhesion; thus, they have the potential to enhance early wound healing around implant and abutment surfaces [25]. Furthermore, positive effects on the proliferation, viability, and spreading of epithelial cells have been demonstrated compared to non-coated (NC) Ti, suggesting a possibility to enhance the attachment of soft tissue to the implant or abutment surface and thereby having the potential to improve the success rate of dental implant treatment [26].

The connective tissue surrounding a dental implant comprises two distinct zones: an inner zone near the implant surface, rich in fibroblasts, and a lateral zone, which contains a higher concentration of collagen fibers and blood vessels [2,27]. Integrins, vinculin, and paxillin are important molecules in fibroblast focal adhesion [28,29]. Integrins penetrate through the cell membrane to the extracellular matrix and are expressed as α2, α4, α5, β1, and β3 subunits [30,31]. Both vinculin and paxillin are localized within the cytoplasm of the cell [28]. Vinculin is an actin-binding protein assumed to play a crucial role in integrin-mediated cell adhesion. It contributes to cell spreading by stabilizing focal adhesions [32]. Furthermore, it links integrins to the actin cytoskeleton, which consists of two domains (head and tail). The head domain binds to actin-binding proteins like talin, while the tail domain interacts with paxillin and actin filamentous (F-actin) [32,33]. Paxillin is essential for the structural organization of focal adhesion sites [29]. It is located at the end of F-actin as it can bind to vinculin [28,29], focal adhesion kinase (FAK), and other proteins. Their binding interactions influence cell behavior and focal adhesion dynamics [34].

The expression of key focal adhesion molecules can indicate the quality of attachment between the connective tissue and the implant surface. Therefore, the current study aims to investigate focal adhesion formation via the expression of vinculin and paxillin alongside cell proliferation of human gingival fibroblasts (HGFs) on ALD-HA-coated Ti surfaces. The hypothesis is that the ALD-HA coating enhances the expression of focal adhesion molecules and promotes HGF cell adhesion and proliferation.

## 2. Materials and Methods

### 2.1. Nanocrystalline Hydroxyapatite-Coated Ti Discs Preparation

The process for preparing ALD-HA coatings has been detailed in a previous study [35]. Briefly, square 50 mm and 1 mm thick Ti plates (Grade 2, ASTM B265 specification (ASTM B265-20a; Standard Specification for Titanium and Titanium Alloy Strip, Sheet, and Plate. ASTM International: West Conshohocken, PA, USA, 2020), William Gregor Ltd., London, UK) were utilized as substrate. The ALD-HA coating was produced by initially depositing a thin ${CaCO}_{3}$ film in an F-120 ALD reactor (ASM Microchemistry Ltd., Helsinki, Finland) using a monomeric deposition precursor (Ca(thd)_2_; Volatec Oy, Porvoo, Finland) and $O_{3}$. The Ca(thd)_2_ was evaporated at 188 °C, while $O_{3}$ was generated from high-purity $O_{2}$ (99.9999%) using an ozone generator (Wedeco Ozomatic Modular 4 HC, Nottingham, UK). The deposition process was carried out at a high temperature (250 °C) over 2000 ALD cycles. Afterward, the obtained ${CaCO}_{3}$ film on the Ti plate was transformed into HA by immersion in a 200 mM $A=\pi r^{2}$ ${(NH}_{4}{)}_{2}{HPO}_{4}$ solution (Merck, 99%, Darmstadt, Germany) at 95 °C. Finally, the plates were thoroughly rinsed with deionized water and then dried with compressed air. The plates are then cut into 7 × 7 mm discs with a manual plate cutter (Bernardo PTS 1050 S, Linz, Austria).

### 2.2. Surface Characterization

The surface topography was evaluated using a scanning electron microscope (SEM; LEO Gemini 1530, Carl Zeiss, Oberkochen, Germany), and an X-ray detector (Thermo Scientific, Waltham, MA, USA) was used for the Energy-Dispersive X-ray Spectroscopy (EDS) analysis. The surface roughness of the NC and ALD-HA discs was assessed with a profilometer (3D non-contact optical; Bruker Nano GmbH, Billerica, MA, USA). The surface profile images were captured using a 5× objective lens and a 0.5× multiplier. The average surface roughness Sa (arithmetic mean height) was calculated using Vision 64 software based on measurements taken at six randomly selected locations on each disc.

### 2.3. Cell Culture

The primary HGFs used in this study were obtained from a gingival biopsy specimen of healthy young adult patients with their third molars surgically extracted (Oral Health Care, City of Turku, Finland). Ethical approval for the collection of gingival biopsy samples was obtained from the Hospital District of Southwest Finland (63/1801/2020). The tissues from periodontally healthy site samples were maintained in cell DMEM (Dulbecco Modified Eagle’s Medium; Thermo Fisher Scientific, Waltham, MA, USA). The DMEM was supplemented with 10% fetal bovine serum, 100 IU/mL penicillin, and 100 µg/mL streptomycin (Gibco BRL, Life Technologies, Paisley, UK). The cells were incubated at 37 °C in 5% ${CO}_{2}$, and the DMEM was changed thrice a week. The HGFs were acquired from passages 13 to 14 and cultivated on the samples at 30,000 cells/cm^2^ density.

### 2.4. Cell Proliferation Assay

The cell proliferation on the NC and ALD-HA discs was assessed using Alamar Blue™ assay (Thermo Fischer, Waltham, MA, USA), based on colorimetric format. HGFs were plated on the Ti discs and cultured for 1, 3, and 7 d (*n* = 12/group/time point). The Ti discs were removed from the DMEM at predetermined times and transferred into sterile culture plates containing fresh DMEM with 10% Alamar Blue reagent. The Alamar Blue solution was then incubated on the Ti discs for 3 h at 37 °C in a 5% ${CO}_{2}$ environment. Subsequently, 150 µL of the solution from each disc was pipetted to calculate the absorbance value with the wavelength of 569 and 594 nm (Multiskan FC, Thermo Scientific). Measured absorbances were used to calculate the reduction of assay reagent, and the cell proliferation rate was measured at different time points for the test (ALD-HA) and the control (NC) groups. The experiment was conducted three times.

### 2.5. Adhesion Protein Analyses

Western blot analysis was conducted to examine the expression of adhesion proteins on ALD-HA-coated and NC Ti discs. HGFs were cultured on Ti discs for 3 d. After incubation, the discs were rinsed once with PBS and lysed using a pre-warmed TXLB buffer (95 °C) (50 mM Tris-HCl (pH 7.5), 0.5% Triton-X, 0.5% glycerol, 150 mM NaCl, 1% SDS, complete protease inhibitor (Sigma-Aldrich, Darmstadt, Germany), and PhosSTOP tablet (Sigma-Aldrich, Darmstadt, Germany)). The cell lysates were collected in Eppendorf tubes incubated at 95 °C for 10 min, followed by storage at −20 °C. The protein concentration of each sample was quantified using the Bio-Rad Protein Assay Reagent. Equal amounts of protein from each sample were mixed with 6x sample buffer and loaded onto Mini Protean TGX Precast SDS-PAGE Gels (Bio-Rad, Hercules, CA, USA). Proteins were then transferred to membranes (Trans-Blot Turbo Transfer System, Bio-Rad). The membranes were rinsed two times with Milli-Q water (mQ) and once with Tris-buffered saline containing Tween (TBST) and then blocked with 5% milk in TBST for 1 h. Subsequently, the membranes were incubated overnight at 4 °C with primary antibodies prepared in 5% milk; (vinculin (1:1000, V9131, Sigma-Aldrich), paxillin (1:5000, 612405, BD Biosciences), and GAPDH (5G4MaB6C5, Hytest, Turku, Finland 1:20,000). The following day, the membranes were rinsed three times with TBST and incubated for 1 h with secondary antibodies (Donkey Anti-Mouse, IRDye 680 RD, LI-COR Biosciences, Bourne, MA, USA, 1:5000). After this, the membranes were rewashed three times with TBST and subsequently imaged using the Li-Cor Odyssey Infrared Imager. Western blotting was performed using three biological replicates.

### 2.6. Immunofluoresence Staining and Confocal Microscopy

To investigate focal adhesion formation and fibroblast cell spreading on Ti discs, cells were cultured on the discs for 2 and 24 h at 37 °C (*n* = 4/group/time point). At each time point, 4% paraformaldehyde was used to fix the discs for 15 min at 37 °C, rinsed with phosphate buffered saline (PBS), and kept at +4 °C thereafter. Then, the discs were permeabilized with 0.5% Triton-X-100 in PBS for 15 min. Primary antibodies (vinculin (V9131, 1:100, Sigma-Aldrich) and paxillin (ab32084, 1:500, Abcam, Cambridge, UK)) were prepared in PBS containing 30% horse serum and incubated with the discs overnight. The following day, the discs were rinsed three times with PBS and then treated with secondary antibodies (Anti-Rabbit, 1:400, A11011, Anti-Mouse, 1:400, A21202 (ThermoFisher Scientific, Waltham, MA, USA) and DAPI (1:200, nucleus staining,) and Phalloidin Atto (1:400, Sigma-Aldrich)) prepared in PBS containing 30% horse serum for 1 h at room temperature. Afterward, the samples were rinsed with PBS and glued onto a microscope glass using Mowiol (Sigma-Aldrich). The stained samples were imaged using a spinning disc confocal microscope equipped with a 63× Zeiss Plan-Apochromat objective (Carl Zeiss Microscopy Deutschland GmbH, Oberkochen, Germany), Hamamatsu sCMOS Orca Flash4.0 camera (Hamamatsu Photonics, Hamamatsu, Japan), and a 3i CSU-W1 Spinning Disk (Intelligent Imaging Innovations, Inc., New York, NY, USA). Adhesion signals from paxillin and vinculin molecules at the bottom cell layer were analyzed using ImageJ Fiji software, Version 1.54p. ImageJ was also used to calculate cell areas and the number and size of focal adhesions in each group. Focal adhesion size, stained with vinculin, was calculated from confocal images, following the method previously described by Horzum et al. [36]. Three biological replicates were performed for each staining.

### 2.7. Statistical Analysis

ImageJ, a Fiji program, was used to analyze Western blot and confocal microscope images. Graphs were created, and data were analyzed using the GraphPad Prism program, version 10.5.0. The unpaired *T*-test and Mann–Whitney test were used to assess the statistical significance of cell spreading, Western blot analyses, cell proliferation assay results, and adhesion protein signals.

## 3. Results

### 3.1. Surface Characteristics

**Figure 1:**
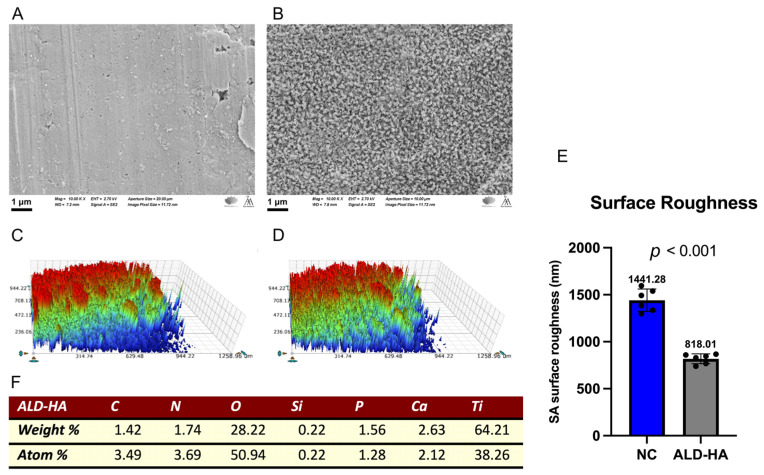
Scanning electron microscopy (SEM) images of non-coated (**A**) and ALD-HA-coated (**B**) titanium discs at 10,000× magnification. Panel (**C**,**D**) show the non-coated and ALD-HA-coated surface profiles, respectively. Panel (**E**) presents the mean and standard deviation of the surface roughness (Sa) values, with a statistically significant difference between groups (*p* < 0.001). The black dots represent individual surface roughness values. Panel (**F**) presents the EDX analysis of the ALD-HA disc surface.

The surface topography of ALD-HA-coated and NC discs was examined using SEM. The NC discs displayed a relatively smooth surface with visible grinding lines (Figure 1A). In contrast, the ALD-HA-coated discs were significantly (*p* < 0.001) smoother and entirely covered with crystalline coatings composed of small, nanoscale HA crystals (Figure 1B). The surface roughness profiles (Sa) of the coated and NC discs are presented in Figure 1C,D. The ALD-HA-coated discs showed significantly (*p* < 0.001) lower surface roughness compared to the NC discs (Figure 1E), resulting in a smoother surface topography in both the X and Y profiles. Figure 1F shows the EDX analysis of the ALD-HA-coated discs. Table 1 shows the contact angle (CA) and surface free energy (SFE) on the NC and ALD-HA-coated discs. CA and SFE data were extracted from a previous publication [25].

### 3.2. Cell Proliferation

**Figure 2:**
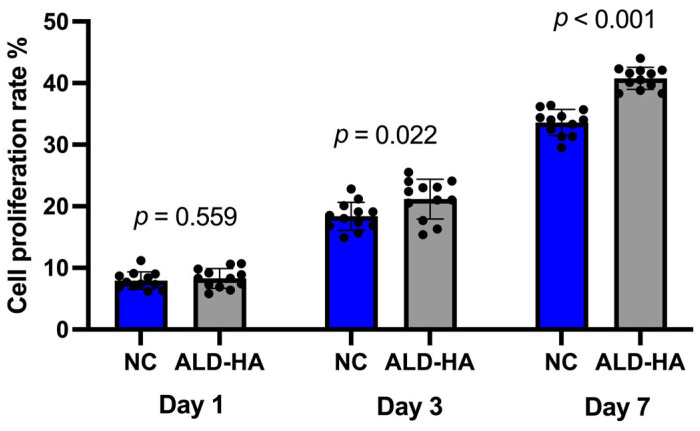
Proliferation of human gingival fibroblasts on non-coated (NC) and ALD-HA-coated titanium discs evaluated after 1, 3, and 7 d of culture. The ALD-HA-coated surfaces exhibited significantly higher cell attachment compared to the non-coated discs at both d3 (*p* = 0.022) and d 7 (*p* < 0.001), the back dots indicate individual values. (*n* = 3 biologically independent experiments).

HGFs were cultured for 7 d, and cell proliferation rate was measured to assess whether cell activity was enhanced on the ALD-HA surface. The cell proliferation on all titanium discs showed a consistent increase over time. The proliferation rate was significantly higher on the ALD-HA surface compared to the NC Ti surface (*p* < 0.05, <0.001) after 3 and 7 d of cell culture, respectively (Figure 2).

### 3.3. Western Blotting

**Figure 3:**
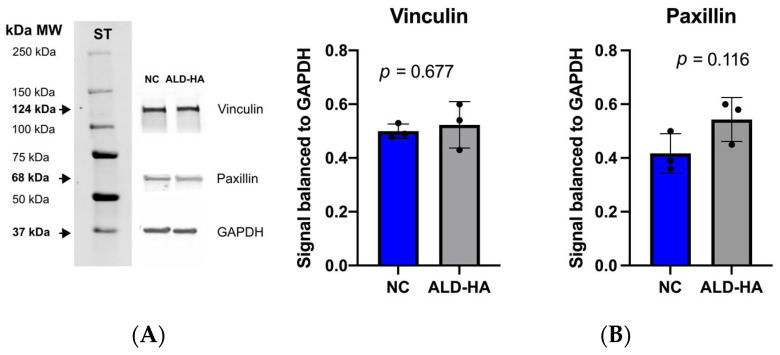
Expression of focal adhesion proteins, vinculin and paxillin, on non-coated and ALD-HA-coated discs. (**A**) Representative Western blotting of vinculin, paxillin, and GAPDH (loading control). ST = the protein standard with labeled molecular weight (MW) markers. (**B**) Paxillin and vinculin signal levels, normalized to GAPDH on non-coated and ALD-HA-coated titanium discs after 3 d of cultivation. There was no statistically significant difference between the non-coated and coated.discs. The black dots indicate the individual values of protein expression. *n* = 3 biological independent experiments.

Western blotting was performed to analyze whether improved cell proliferation would indicate higher adhesion protein expression. After 3 d of cell culture, the vinculin level was more comparable between coated and NC surfaces, whereas the level of paxillin was slightly higher on the ALD-HA surface compared to the NC surface. However, no statistically significant difference was found in paxillin level between ALD-HA-coated and the NC Ti surfaces (Figure 3).

### 3.4. Confocal Microscope Analysis

#### 3.4.1. Cell Spreading

**Figure 4:**
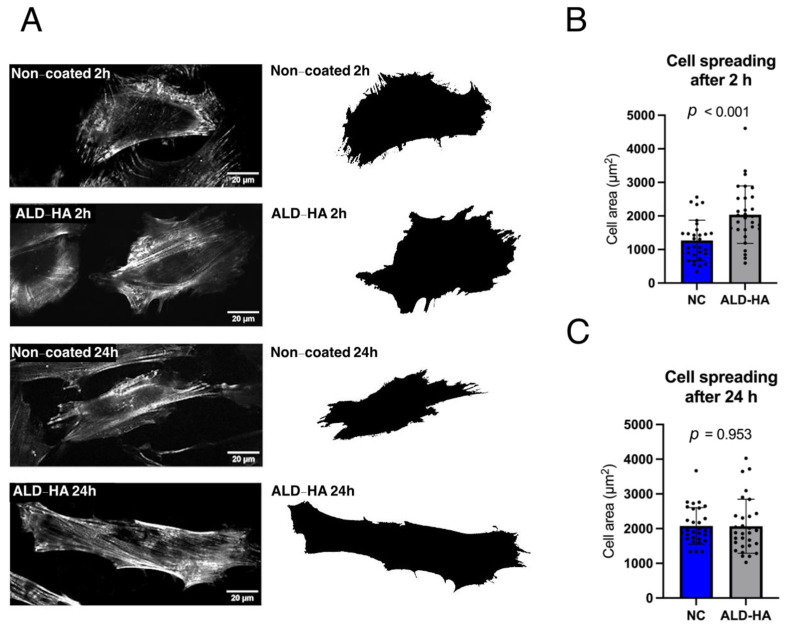
Cell spreading on non-coated and ALD-HA-coated titanium discs. Panel (**A**) illustrates representative images showing the actin cytoskeleton and overall cell morphology at 2 and 24 h. Panels (**B**,**C**) present the quantified cell area at 2 and 24 h, respectively. Data represent mean ± SD. The black dots indicate the number of measured cells, (*n* = 30 cells). A statistically significant difference in cell area was observed at the 2 h time point (*p* < 0.001).

High-resolution confocal microscopy and subsequent image analysis were conducted to assess whether the enhanced cell proliferation on ALD-HA coatings was correlated with increased cell spreading (Figure 4A). The approximate spread areas of attached HGF cells were quantified from the confocal images. After 2 h, fibroblast spreading was significantly greater (*p* < 0.001) on ALD-HA surfaces compared to NC surfaces (Figure 4B). However, after 24 h, no differences in cell spreading were detected between the groups (Figure 4C), suggesting that ALD-HA improves the kinetics of initial cell adhesion.

#### 3.4.2. Focal Adhesion Size and Number

**Figure 5:**
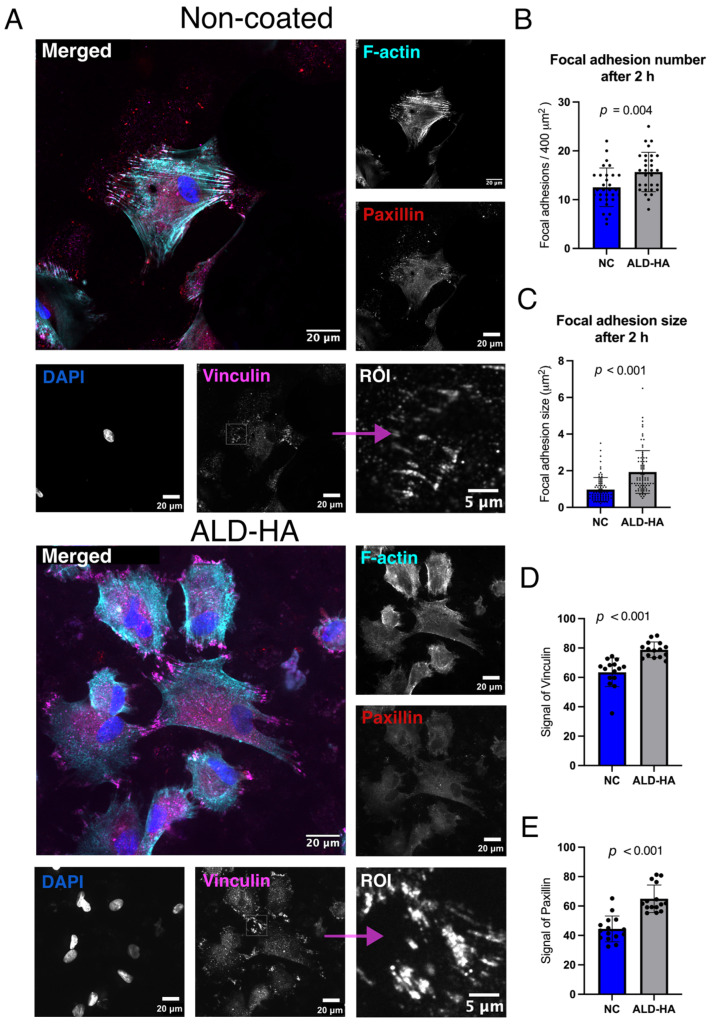
Gingival fibroblasts are more spread with faster focal adhesion formation, as shown on ALD-HA-coated surfaces compared to NC Ti discs. (**A**) Representative images obtained by confocal microscope from the bottom layer of HGFs after 2 h of cultivation. The expression of vinculin and paxillin stained with F-actin, and DAPI as a nucleus. Region of interest (ROI) areas were selected from the the areas of each image, where high expression of focal adhesions were detected, (imaged with 63× Zeiss Plan Apochromat, 3i CSU-W1 Spinning Disk, and Hamamatsu sCMOS Orca Flash4.0 camera). (**B**) The number of focal adhesions per area (400 μm^2^) on coated and NC surfaces after 2 h. (**C**) Size of focal adhesions on surfaces after 2 h. Signal intensities of (**D**) vinculin and (**E**) paxillin on the bottom layer of HGF on coated and NC titanium surfaces after 2 h. Confocal microscope images were done with three biological replicates. Data represent mean ± SD. The balck dots indicate the individual values. Significant *p*–values are marked in the figures.

**Figure 6:**
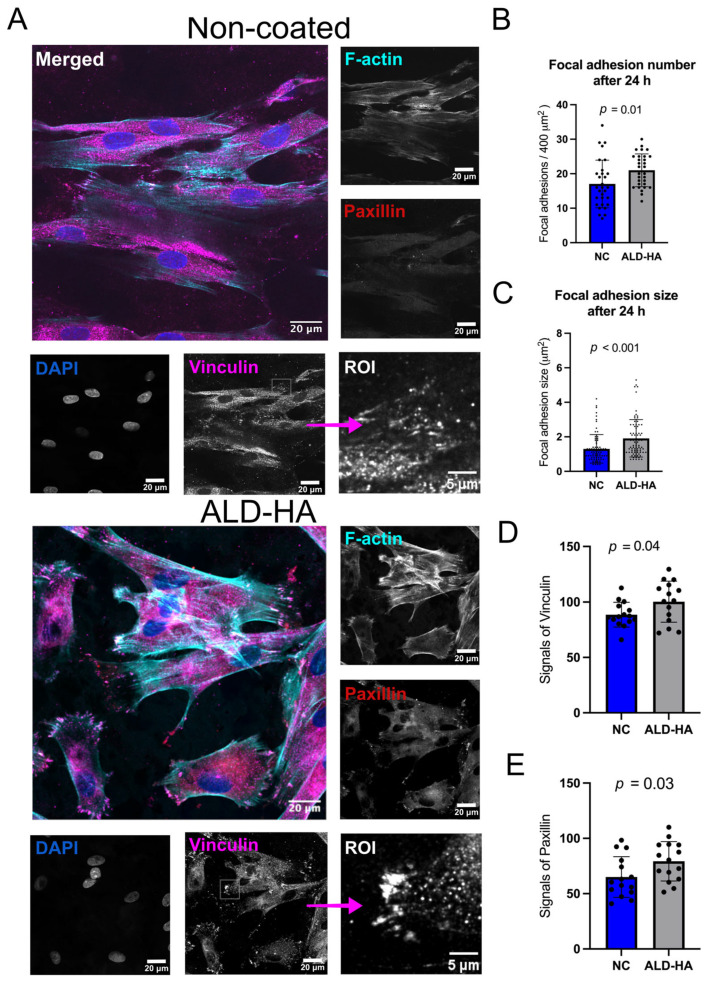
More focal adhesions with larger sizes on ALD-HA-coated surfaces compared to NC titanium discs. (**A**) Images captured via confocal microscopy of HGFs after 24 h of cultivation stained with vinculin, paxillin, F-actin, and DAPI. ROI (region of interest) (imaged with 63× Zeiss Plan Apochromat, 3i CSU-W1 Spinning Disk, and Hamamatsu sCMOS Orca Flash4.0 camera). (**B**) The number of focal adhesions per area (400 μm^2^) on coated and NC surfaces after 24 h of cell culture. (**C**) Focal adhesion size on surfaces after 24 h. Signal intensities of (**D**) vinculin and (**E**) paxillin on the bottom layer of HGF on coated and NC titanium surfaces after 24 h. Data represent mean ± SD. The black dots indicate the individual values. Significant *p*-values are indicated in the figures. Confocal microscopy was performed using three biological replicates.

The number of focal adhesions per area and their size were quantified for each surface to determine if improved cell viability on ALD-HA surfaces correlates with actual focal adhesion formation. Figure 5A and Figure 6A show confocal microscopy images representing focal adhesion protein signals of HGF cells after 2 and 24 h, respectively. These proteins were primarily distributed at focal adhesion sites. The focal adhesions were calculated based on vinculin expression. The number of focal adhesions per area was significantly higher on ALD-HA-coated than on NC controls at 2 h (*p* = 0.004) and 24 h (*p* = 0.01) (Figure 5B and Figure 6B). Focal adhesion sizes on ALD-HA-coated surfaces were also significantly (*p* < 0.001) larger at both time points on the ALD-HA than those on NC discs (Figure 5C and Figure 6C). Additionally, the signal levels of paxillin and vinculin in the bottom layer were significantly higher (*p* < 0.001) after 2 h on the ALD-HA-coated surfaces compared to the NC surfaces (Figure 5D,E). Furthermore, after 24 h, the signal levels of vinculin (*p* = 0.04) and paxillin (*p* = 0.03) also remained significantly higher on the coated than on the NC surfaces (Figure 6D,E).

## 4. Discussion

This study aimed to assess the impact of ALD-HA coating on HGF proliferation, adhesion molecule expressions, and cell spreading. The results demonstrated enhanced cell viability on ALD-HA-coated surfaces, with a significantly higher HGF proliferation rate observed at 3 and 7 d compared to NC Ti. In addition, HGF cells exhibited wide spreading and higher expression of focal adhesion molecules on ALD-HA surfaces at 2 and 24 h time points compared to the NC surfaces. Notably, vinculin and paxillin signals were higher on the focal adhesion area on the coated surfaces. Moreover, the size and number of focal adhesions increased significantly on coated surfaces compared to their NC counterparts. The extent of cell spreading is related to the size of focal adhesions, as demonstrated in a previous study [37]. Therefore, the ALD-HA coating seemed to provide favorable conditions for initial cell adhesion and spreading. These findings are encouraging and support the initial hypothesis.

Nanoscale modification of Ti implant surfaces can alter their chemistry and topography, potentially affecting how these surfaces interact with ions, proteins, and cells [38,39]. These changes stimulate molecular and cellular responses and enhance healing between the implant and surrounding tissues [40,41]. Previous studies have demonstrated that nanostructured coatings featuring smooth topography enhance the activity of fibroblasts and epithelial cells more effectively than machined surfaces, thereby accelerating and improving the quality of tissue regeneration [42,43,44]. The results of this study demonstrate that coating Ti discs with ALD-HA reduces their surface roughness and results in a smoother surface compared to the NC surface.

The wettability of implant materials is a key surface characteristic that affects the biological response of the implant and serves as a predictive indicator for cytocompatibility. Good wettability promotes protein adhesion and enhances the initial cell response [45]. Surfaces with a CA less than 90° are generally considered hydrophilic, whereas those with a CA greater than 90° are categorized as hydrophobic. The ALD surface has a water CA of 76.13°, indicating a more hydrophilic surface than 84.65° for the NC surfaces. Surfaces with hydrophilic properties, such as on the ALD-HA coating, are primarily associated with higher wettability and a favorable cellular response [25,26]. The surface SFE consists of two components: polar (γp) and dispersive (γd). The polar component influences cell behavior, as polar forces primarily govern cell–material interactions [46]. Surfaces with a high polar component of SFE typically exhibit low CA when in contact with polar liquids such as water [47,48]. The ALD-HA surfaces demonstrated a higher polar component compared to the NC surface, which is another factor that can explain the observed favorable fibroblast cell responses. In addition, the presence of calcium and phosphate elements on the ALD-HA-coated surfaces may also contribute to enhanced cell attachment. The deposition of Ca and P on Ti surfaces has been shown to improve biocompatibility and enhance protein adsorption, which, in turn, induces the formation of mucosal attachment [19,49].

In this study, paxillin and vinculin expression levels were significantly elevated on ALD-HA-coated Ti surfaces compared to the NC surfaces. The high expression of these adhesion proteins correlates with actual focal adhesion formation. The size and number of focal adhesions were more significant on the ALD-HA surface than on the NC surface at 2 and 24 h time points. These molecules were located peripherally at focal adhesion spots. As vinculin and paxillin are essential adhesion markers, induced expression of these molecules indicates enhanced cell adhesion to the Ti surface. Findings from this study are consistent with a previous study by Riivari et al., which demonstrated that HGF exhibited greater spreading and increased expression of focal adhesion molecules on ${TiO}_{2}$-coated Ti surfaces [50]. Additionally, vinculin and paxillin signals at focal adhesion sites were more pronounced on ${TiO}_{2}$-coated surfaces than on NC surfaces, indicating higher initial cell adhesion and the potential of such coatings to improve mucosal attachment [50]. However, in blot analyses, no significant differences were found in paxillin and vinculin expression between coated and NC Ti surfaces.

Several studies have shown that, during the early stages of healing, modified abutment surfaces seem to influence cell attachment and the healing process at the Ti–tissue interface [51,52,53]. Generally, collagen fibers align parallel to implant surfaces, which tends to lower resistance to bacterial invasion. However, recent studies have demonstrated collagen fibers oriented perpendicular or obliquely to the modified transmucosal abutment surface [54,55]. Furthermore, rod-like attachments of gingival collagen fibers were seen perpendicularly oriented on the laser-treated Ti implant surface at 6 weeks, leading to improved connective tissue attachment. In contrast, no such attachments were detected on the untreated Ti implant surface [56]. In addition to the previously reported promising effects of the ALD-HA coating on blood and platelet responses [25], it has also been shown to promote higher epithelial cell spreading and increased laminin γ2 expression compared to NC surfaces, indicating good cell adhesion properties [26]. The findings of the current study align with these earlier results, showing greater cell spreading and higher expression of focal adhesion molecules, indicating better adhesion properties on coated surfaces than on NC surfaces. The expression of focal adhesion molecules of paxillin and vinculin may enhance the quality of connective tissue attachment to the implant surface.

While the HA coating produced by the ALD method demonstrates promising advantages, it is important to highlight that this study was conducted under in vitro conditions. Although these conditions provide valuable insights into cellular behavior and material interactions, they do not fully replicate the complexity of the physiological environment. Additionally, the stability of the coating at the soft tissue–implant interface warrants further investigation. Thus, studies evaluating the long-term stability of the coating under physiological conditions are necessary. Nonetheless, even if coating resorption occurs, a sufficiently effective interface may still develop between the Ti and the surrounding peri-implant tissues during this duration. Despite these limitations, findings from the current study indicate that the ALD-HA nanostructured coating promotes HGF adhesion, proliferation, and spreading on Ti surfaces. This emphasizes the need for in vivo studies to assess its impact on epithelial and connective tissues, which will be the focus of our future research.

## 5. Conclusions

The nanoscale crystalline hydroxyapatite coating produced by atomic layer deposition enhances the expression of gingival fibroblast adhesion proteins, promotes focal adhesion formation, and promotes cell spreading on Ti surfaces. In addition, the ALD-HA surface promotes cell proliferation compared to the non-coated surfaces. Improved gingival cell attachment may help reduce the risk of peri-implant infections and enhance the long-term success of implant treatments.

## Figures and Tables

**Table 1:** Mean and standard deviation of water contact angle (°) and surface free energy (TOT; total, D; dispersive, and P; polar) determination by Owens and Wendt (OW) approach on the non-coated and ALD-HA-coated surfaces. * *p* < 0.001. Data were extracted from a previous publication [25].

Sample	Water Contact Angle	Surface Free Energy		
		OW ^TOT^	OW ^D^	OW ^P^
NC	84.65° (4.17)	36.84 (1.12)	32.52 (1.28)	4.55 (0.94)
ALD-HA	76.13° (2.41) *	35.03 (2.08)	27.01 (1.54) *	8.01 (1.10) *

## Data Availability

Data associated with this research is available from the corresponding author upon reasonable request.
